# Effect of a tadalafil 5-mg single daily dose on lifelong premature ejaculation: A single-blinded placebo-controlled study

**DOI:** 10.1080/2090598X.2021.2007464

**Published:** 2022-02-26

**Authors:** Amr Hassan Abou Faddan, Hisham Diab Gaber, Daniar Oamonov

**Affiliations:** a Department of Urology, Faculty of Medicine, Assiut University, Assiut, Egypt; b Department of Dermatology and Andrology, Faculty of Medicine, Assiut University, Assiut, Egypt; c Departmant of Urology and Pediatric Urology, Universitatsklinikum Schleswig-Holstein Campus Kiel, Kiel, Germany

**Keywords:** Premature ejaculation, lifelong premature ejaculation, tadalafil, PEDT, PDE5 inhibitors

## Abstract

**Objective:**

To investigate the effectiveness and safety of a tadalafil 5-mg single daily dose in the treatment of lifelong premature ejaculation (PE).

**Patients and Methods:**

A total of 55 men with lifelong PE were enrolled in this study. The intravaginal ejaculatory latency time (IELT) and Premature Ejaculation Diagnostic Tool (PEDT) were used to assess the efficacy of tadalafil. Baseline recording of the IELT (using a stopwatch) and PEDT was done. Patients were randomised to take a single daily dose of tadalafil 5 mg or placebo. Re-assessment was done by IELT and PEDT after 1 and 3 months.

**Results:**

As regard, age, number of children, frequency of intercourse and duration of PE there are no statically significant differences between both groups. In both the tadalafil 5-mg and placebo groups the IELT statically significantly increased after 1 month by a mean (SD) of 4.8 (4.7) and 5 (4.9) s, and after 3 months by a mean (SD) of 6.6 (7) and 4.5 (9) s; and the PEDT scores significantly improved after 1 month by a mean (SD) of 2.1 (1.9) and 2 (1.5), and after 3 months by a mean (SD) of 3.5 (2.5) and 3.3 (2.2). However, there is was no statically significant difference between the tadalafil 5-mg and placebo groups for the IELT and PEDT at baseline or after 1 and 3 months of treatment.

**Conclusions:**

Despite 5-mg tadalafil once daily being safe and it showed only a slight improvement in IELT and PEDT in the treatment of lifelong PE, failing to show any statistically significant difference from the placebo drug.

**Abbreviations**: ED: erectile dysfunction; 5-HT: 5-hydroxytryptamine (serotonin); IELT: the intravaginal ejaculation latency time; ISSM: International Society of Sexual Medicine; PE: premature ejaculation; PEDT: Premature Ejaculation Diagnostic Tool; PDE5i: phosphodiesterase-5 inhibitor; SSRI: selective serotonin reuptake inhibitor

## Introduction

Premature ejaculation (PE) is a frequent complaint that can have a major negative impact on both partners. As PE is linked to lower satisfaction, it leads to less sexual intercourse [1,2]. PE is the most common sexual disorder, affecting up to 30% of men worldwide [3].

PE is described as ‘a male sexual dysfunction characterised by emission that always or nearly always occurs prior to or within about 1 minute of vaginal penetration (lifelong PE) OR a clinically relevant reduction in latency time, sometimes to about 3 minutes or less’ by the International Society of Sexual Medicine (ISSM) (acquired PE) and the inability to postpone ejaculation on all or almost all vaginal penetrations, as well as negative personal effects such as pain, bother, irritation, and/or sexual contact avoidance [4].

Premature ejaculation is characterised as ‘a constant or repeated pattern of ejaculation occurring during partnered sexual activity within approximately 1 minute following vaginal penetration and before the person wishes it’ in Diagnostic and Statistical Manual of Mental Disorders, fifth edition (DSM-5), but lifelong PE is not separately defined [5].

The underlying cause of this condition is still unknown. PE was once thought to be caused by glans penis hyperesthesia, irregular local anatomy of the urogenital system, or psychosomatic anomalies [6]. However, there has been increasing evidence in recent years for underlying genetic and neurological mechanisms. The serotonin (5-hydroxytryptamine [5-HT]) 1A or 2 C receptor genes, as well as the dopamine transporter gene, may, for example, contribute to the intravaginal ejaculation latency time (IELT) of lifelong PE, although this remain debatable [7,8].

Furthermore, Waldinger et al. [9] hypothesised in 1998 that lifelong PE in the form of an IELT of <1 min is associated with genetic factors, decreased central 5-HT neurotransmission, hyperfunction of 5-HT1A receptors, and hypofunction of 5-HT2C receptors.

In order to overcome all these facets of PE a successful therapy needs to enhance both the IELT and subjective outcomes, such as perceived lack of control, distress, and interpersonal difficulties, to address all the aspects of PE [10].

Multiple treatment modalities, including topical anaesthetics, α-adrenergic receptor antagonists, tricyclic antidepressants, selective serotonin-reuptake inhibitors (SSRIs), and behavioural methods, have been tried for PE. In recent years, phosphodiesterase-5 inhibitors (PDE5i), which are primarily used to treat erectile dysfunction (ED), have been used in some clinical trials for the treatment of PE.

Tadalafil is a PDE5i that is accepted for on-demand dosing in the treatment of ED, as well as once-daily dosing in the treatment of ED or signs and symptoms of BPH or a combination of both [11].

In the present study, we attempted to investigate the efficacy and safety of a tadalafil 5-mg single daily dose in the treatment of lifelong PE.

## Patient and methods

From June 2019 to December 2020, 227 patients were evaluated who complained of PE sought medical counselling at the ambulatory clinic of the Urology and the Andrology departments at Assiut University Hospital. For study inclusion patients had to fulfil the lifelong PE criteria according to the ISSM 2014, with ejaculation taking place at ≤1 min of vaginal penetration, failure to defer ejaculation and lack of sexual intimacy leading to adverse personal outcomes. In addition, the patients had to be aged between 20 and 35 years, with average body mass index, married with stable sexual relationship with an intercourse frequency of two to three times per week for at least a month before the study. The patient also had to have complained of PE since his initiation of sexual activity, which was ≥6-months duration, with good erections (five-item version of the International Index of Erectile Function [IIEF-5] score of ≥22), not hypertensive or diabetic or have any comorbidities, and not receiving any medication.

**Figure 1.**
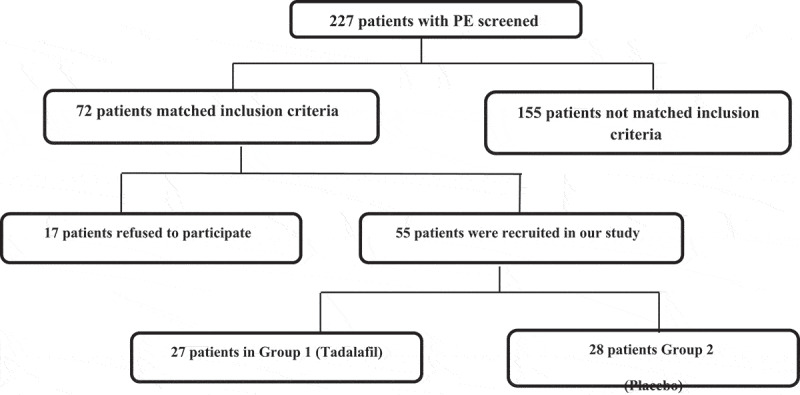
Flow chart of the patients included in the study.

Apart from routine medical history, clinical and genital examination, at baseline the Premature Ejaculation Diagnostic Tool (PEDT) was completed and the IELT was calculated (using a stopwatch) for all patients to evaluate ejaculatory function. In all, 72 patients matched our inclusion criteria; however, 17 patients refused to participate in this study, hence 55 patients were recruited to the single-blind placebo-controlled clinical study (Figure 1). As none of the patients knew the essence of the medications they were given, they were randomly divided into two groups using a coin toss. Tadalafil 5 mg was received daily in the first group and placebo in the second group was given for 3 months. The patients were evaluated after 1 and 3 months by PEDT and IELT. Well informed agreement was obtained from the included patients after explaining the pro and cons of this research. Refusal to participants did not undermine the provided medical care.

## Results

The mean (SD) age of the tadalafil 5-mg group was 30.4 (3.8) years and was 30.4 (3.8) years in the placebo group. In both groups ~50% were smokers and had been married for >5 years with regular sexual intercourse about two times per week and had one to two children. The mean (SD) baseline IELT in tadalafil 5-mg group was 45.7 (9.1) s, while in the placebo group it was 43.2 (12.1) s (*P* = 0.127), and the mean (SD) baseline PEDT score was 14.7 (2.4) and 14.2 (3.2) in tadalafil 5-mg and placebo groups, respectively (*P* = 0.128; Table 1).

**Table 1.** Baseline characteristics of tadalafil 5-mg and placebo patients

	Tadalafil 5 mg (*N* = 27)	Placebo (*N* = 28)	P*
Age, years, mean (SD)	30.4 (3.8)	30.4 (3.8)	0.941
Smoker, *n* (%)	14 (51.9)	14 (50)	0.553
Duration of PE, years, mean (SD)	5.8 (2)	5.9 (2.4)	0.109
Number of children, mean (SD)	2.1 (1)	1.9 (1.1)	0.705
Frequency of intercourse/week, mean (SD)	2.6 (0.9)	2.4 (0.9)	0.653
Baseline IELT, s, mean (SD)	45.7 (9.1)	43.2 (12.1)	0.127
Baseline PEDT score, mean (SD)	14.7 (2.4)	14.2 (3.2)	0.128

*Independent *t*-test.

The mean IELTs increased from baseline to the 1-month follow-up in both groups; however, there was no statistically significant difference between the groups, with a mean (SD) improvement of 4.8 (4.7) and 5 (4.9) s (*P* = 0.657), and this was the similar at 3 months, with an improvement of 6.6 (7) and 4.5 (9) s (*P* = 0.610) (Table 2).

**Table 2.** Comparisons of the mean (SD) IELTs and total PEDT scores between the tadalafil 5-mg and placebo groups after 1 and 3 months

Variable, mean (SD)	Tadalafil 5 mg (*N* = 27)	Placebo (*N* = 28)	P*
IELT after 1 month, s	50.6 (9.3)	48.2 (10)	0.772
IELT after 3 months, s	52.3 (7.6)	47.7 (9.6)	0.153
Improvement in IELT after 1 month, s	4.8 (4.7)	5 (4.9)	0.657
Improvement in IELT after 3 months, s	6.6 (7)	4.5 (9)	0.610
PEDT score after 1 month	12.6 (2.8)	12.2 (2.9)	0.852
PEDT score after 3 months	11.2 (3.2)	10.7 (2.8)	0.358
PEDT improvement after 1 month	2.1 (1.9)	2 (1.5)	0.713
PEDT improvement after 3 months	3.5 (2.5)	3.3 (2.2)	0.812

*Independent *t*-test.

The present study did not demonstrate statistically meaningful discrepancies in the mean value of all questions and overall PEDT scores after 1 or 3 months of therapy between tadalafil 5-mg patients and those taking placebo (Tables 3–5). Five patients (18.5%) in the tadalafil 5-mg group complained of headaches, while only two patients complained of back pain and myalgia (Table 6).

**Table 3.** PEDT scores at baseline

No.	Question	Tadalafil 5 mg (*N* = 27), mean (SD)	Placebo (*N* = 28), mean (SD)	*P*
1	How difficult is it for you to delay ejaculation?	2.9 (1)	2.6 (1.2)	0.382
2	Do you ejaculate before you want to?	3.2 (0.7)	3 (1)	0.169
3	Do you ejaculate with very little stimulation?	2.4 (1.1)	2.2 (1.3)	0.279
4	Do you feel frustrated because of ejaculating before you want to?	2.7 (1)	3 (1.1)	0.866
5	How concerned are you that your time to ejaculation leaves your partner unfulfilled?	2.9 (1.1)	2.7 (1.2)	0.177
Total score		14.7 (2.4)	14.2 (3.2)	0.128

**Table 4.** PEDT scores after 1 month

No.	Question	Tadalafil 5 mg (*N* = 27), mean (SD)	Placebo (*N* = 28), mean (SD)	*P*
1	How difficult is it for you to delay ejaculation?	2.9 (0.8)	2.7 (1)	0.1
2	Do you ejaculate before you want to?	2.5 (0.9)	2.6 (0.8)	0.495
3	Do you ejaculate with very little stimulation?	2.6 (0.9)	2.5 (0.9)	0.993
4	Do you feel frustrated because of ejaculating before you want to?	2.4 (0.7)	2.3 (0.7)	0.553
5	How concerned are you that your time to ejaculation leaves your partner unfulfilled?	2.3 (0.7)	2.1 (0.9)	0.731
Total score		12.6 (2.8)	12.2 (2.9)	0.852

**Table 5.** PEDT scores after 3 months

No.	Question	Tadalafil 5 mg (*N* = 27)	Placebo (*N* = 28)	*P*
1	How difficult is it for you to delay ejaculation?	2.7 (1.1)	2.4 (1)	0.326
2	Do you ejaculate before you want to?	2.3 (0.9)	2.1 (0.8)	0.181
3	Do you ejaculate with very little stimulation?	2.2 (1.1)	2 (1)	0.264
4	Do you feel frustrated because of ejaculating before you want to?	2.1 (0.6)	2.2 (0.9)	0.055
5	How concerned are you that your time to ejaculation leaves your partner unfulfilled?	1.9 (0.6)	2 (0.8)	0.570
Total score		11.2 (3.2)	10.7 (2.8)	0.358

**Table 6.** Drug-related side-effects

Side-effect, *n* (%)	Tadalafil 5 mg (*N* = 27)	Placebo (*N* = 28)	*P*
Headache	5 (18.5)	0	0.005
Back pain and myalgia	2 (7.4)	0	
Flushing	3 (11.1)	0	
Total	10 (37)	0	

Both groups showed statically significant improvements in total PEDT scores and prolonged IELT after 1 and 3 months; however, there was no statistical difference between the tadalafil 5-mg and placebo groups.

## Discussion

Over the last 10 years, the pathophysiology of lifelong PE has not been clearly identified [12]. For lifelong PE, one must have this complaint since the first sexual encounter and almost every intercourse; thus, there may be a possible genetic reason in the aetiology of lifelong PE [6].

Atalay et al. [13] looked at the macro- and microstructural variations in the brains of patients with lifelong PE. Their findings revealed that the caudate nucleus, a microstructural component of the basal ganglia, was substantially larger in patients with lifelong PE than in healthy individuals, and that there was a substantial positive relationship between caudate volume and PEDT score, but a negative relationship between IELT, which measures the severity of symptoms, and caudate nucleus volume.

Lifelong PE is largely dependent on medications, most of which are used off-label with the exception of dapoxetine. Local anaesthetic agents and SSRIs have repeatedly shown their effectiveness in the management of lifelong PE. Furthermore, these drugs have unknown long-term consequences [14]. Many guidelines and the United States Food and Drug Administration (FDA) have approved PDE5i for the treatment of ED, but not PE [15].

The role of the PDE5i in the treatment of PE is questionable. Some studies show the use of PDE5i on-demand, including tadalafil, may help in the treatment of PE [16]. Mattos et al. [15] speculated that PDE5i could act in the nitric oxide/cyclic GMP transduction peripherally through involuntary muscle relaxation in the prostate, seminal vesicles, vas deferens, and urethral smooth muscle and centrally through reducing central sympathetic output and refractory time after orgasm. PDE5i can improve erection and self-confidence, but to what extent tadalafil intervention could potentially extend the time to ejaculation has not been ascertained [17].

The majority of the other research proposed that PDE5i could be used alone or in combination with other treatments to treat lifelong PE, but these studies had substantial methodological flaws. For example, in the Aversa et al. [17] study the insufficiency of randomisation, the absence of a placebo arm, and a certain degree of registration distraction (24% of patients with secondary PE and no measurement of pre-potency) reflect major research shortcomings. Besides that, the Abu El-Hamd [16] study was not controlled by a placebo and potency was not objectively measured. In our present sample, we selected tadalafil 5-mg and placebo patients randomly from patients who scored ≥22 on the in IIEF-5.

Notably, PDE5i improve and prolong erection, affecting treatment secondary PE; however, they have many side-effects, but not lifelong PE. There is therefore no effectiveness of sildenafil and tadalafil [15]. In our present research, IELT and PEDT improvements were seen in both the tadalafil 5-mg and placebo cohorts, while the disparity between the drugs and placebo was not statistically significant.

According to Atan et al. [18] PDE5i have no effect on IELT in men with lifelong PE, as they found no substantial ‘cure’ or ‘improvement’ difference between men treated with sildenafil (40% vs 55%, *P* > 0.05) and men treated with placebo. Also, Mondaini et al. [19] have described only subtle changes in IELTs between sildenafil- and placebo-treated groups. Ultimately, sildenafil has not been shown to be effective in PE treatment.

While both tadalafil and placebo dramatically improved IELT and PEDT, after 1 and 3 months of therapy for men with lifelong PE, no substantial difference was seen between the groups. The improvement with tadalafil and placebo found in patients with lifelong PE in our present study is possibly due to enhance erection, self-worth, and ejaculatory control even in men without ED; and by reducing anxiety and post-ejaculatory refractory time, as observed by the increases from baseline IELT and PEDT in these men. However, a multicentre, larger sample would be essential to prove a minor role of tadalafil for everyday use in patients with lifelong PE.

## Data Availability

The data confidentiality was ensured. Data that support the findings of this study are available on request from the corresponding author. The data are not publicly available due to privacy or ethical restrictions.
